# Cost-effectiveness of human papillomavirus vaccination for prevention of cervical cancer in Taiwan

**DOI:** 10.1186/1472-6963-10-11

**Published:** 2010-01-11

**Authors:** Pang-Hsiang Liu, Fu-Chang Hu, Ping-Ing Lee, Song-Nan Chow, Chao-Wan Huang, Jung-Der Wang

**Affiliations:** 1 National Clinical Trial and Research Center, National Taiwan University Hospital, Taipei, Taiwan; 2 Institute of Occupational Medicine and Industrial Hygiene, College of Public Health, National Taiwan University, Taipei, Taiwan; 3 Department of Pediatrics, National Taiwan University Hospital, Taipei, Taiwan; 4 Department of Obstetrics & Gynecology, National Taiwan University Hospital and College of Medicine, National Taiwan University, Taipei, Taiwan; 5 Departments of Internal Medicine and Environmental and Occupational Medicine, National Taiwan University Hospital, Taipei, Taiwan

## Abstract

**Background:**

Human papillomavirus (HPV) infection has been shown to be a major risk factor for cervical cancer. Vaccines against HPV-16 and HPV-18 are highly effective in preventing type-specific HPV infections and related cervical lesions. There is, however, limited data available describing the health and economic impacts of HPV vaccination in Taiwan. The objective of this study was to assess the cost-effectiveness of prophylactic HPV vaccination for the prevention of cervical cancer in Taiwan.

**Methods:**

We developed a Markov model to compare the health and economic outcomes of vaccinating preadolescent girls (at the age of 12 years) for the prevention of cervical cancer with current practice, including cervical cytological screening. Data were synthesized from published papers or reports, and whenever possible, those specific to Taiwan were used. Sensitivity analyses were performed to account for important uncertainties and different vaccination scenarios.

**Results:**

Under the assumption that the HPV vaccine could provide lifelong protection, the massive vaccination among preadolescent girls in Taiwan would lead to reduction in 73.3% of the total incident cervical cancer cases and would result in a life expectancy gain of 4.9 days or 8.7 quality-adjusted life days at a cost of US$324 as compared to the current practice. The incremental cost-effectiveness ratio (ICER) was US$23,939 per life year gained or US$13,674 per quality-adjusted life year (QALY) gained given the discount rate of 3%. Sensitivity analyses showed that this ICER would remain below US$30,000 per QALY under most conditions, even when vaccine efficacy was suboptimal or when vaccine-induced immunity required booster shots every 13 years.

**Conclusions:**

Although gains in life expectancy may be modest at the individual level, the results indicate that prophylactic HPV vaccination of preadolescent girls in Taiwan would result in substantial population benefits with a favorable cost-effectiveness ratio. Nevertheless, we should not overlook the urgency to improve the compliance rate of cervical screening, particularly for older individuals.

## Background

Cervical cancer is one of the most common female malignancies worldwide. The cervical cancer rate has declined in Taiwan over the last decade, an effect largely attributed to widespread screening for cervical cancer. Nonetheless, the compliance with cervical screening in Taiwan remains suboptimal that the annual screening rate was 28.6% for women aged over 30 years [1], and the incidence of cervical cancer is consistently higher than those in neighboring countries [2]. In 2006, there was an annual incidence rate of 16.2 per 100,000 people for invasive cervical cancer and a mortality rate of 7.8 per 100,000 people in comparison with breast cancer incidence and mortality of 61.1 and 12.8 per 100,000 people, respectively [3].

Genital infection with human papillomavirus (HPV) has been well established to be the determining cause of cervical cancer [4,5]. Researchers reported the HPV prevalence in Taiwan was around 10-20% [6-9]. While HPV comprises a wide range of genotypes, several types are defined as high-risk, or oncogenic, for their strong carcinogenicity. A primary preventive measure involving prophylactic vaccination against these oncogenic HPVs has thus been developed, and there are two vaccines that are currently available. One is the bivalent vaccine [10,11], and the other is the quadrivalent vaccine [12], which commonly target the HPV-16 and HPV-18. Safety and satisfactory efficacy against type-specific HPV infection and related precancerous lesions have been demonstrated for both vaccines. Although their efficacy for preventing cervical cancer has not been comprehensively proven yet, it seems reasonable to expect such an outcome.

A number of studies have been conducted to evaluate the potential cost-effectiveness for prevention of cervical cancer through HPV vaccination, with a range of results [13-26]. Indeed, the cost-effectiveness of HPV vaccination varies between regions by many factors including different epidemiology of HPV infection and cervical screening efforts; Puig-Junoy and Lopez-Valcarcel reported that large variations existed in the cost-effectiveness results of different studies even for the same country [27]. Currently, there are still limited data evaluating the economic impact of cervical cancer vaccination in Taiwan [28,29]. The aim of this study was therefore to assess the cost-effectiveness of prophylactic HPV vaccination on the prevention of cervical cancer in Taiwan.

## Methods

### Decision model

**Figure 1:**
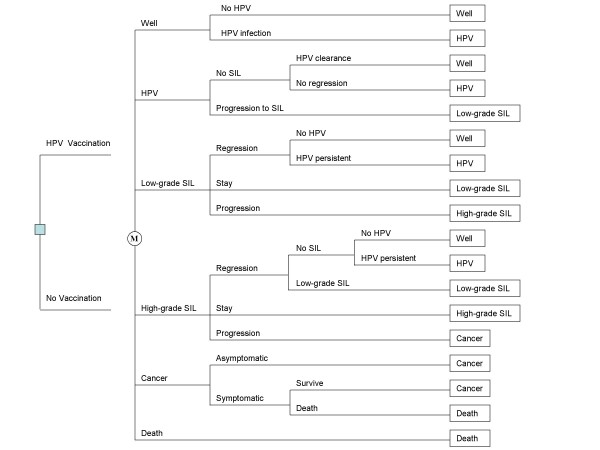
**The Markov decision model**. The square on the left represents the prophylactic vaccination decision. Each woman's health is tracked by a Markov model after entering the Markov tree (denoted by circles containing an alphabet 'M'). In each cycle, women are at risk of developing oncogenic human papillomavirus (HPV) infection, SIL (squamous intraepithelial lesions), cervical cancer or mortality. The Markov tree branches with ends of rectangles represent the above clinical events that can occur during each 1-year period as a 12-year-old girl is followed until death.

We developed a Markov model [30] to assess the cost-effectiveness of the prophylactic vaccine against high-risk HPV infections and related cervical cancers in Taiwan (Figure 1) using the TreeAge software (TreeAge Software, Inc., Williamstown, MA, USA). The perspective of analysis considered in this study was that of the healthcare payers. The target population for our analysis included all adolescent girls in Taiwan; the time horizon was lifetime.

Our model simulated the natural history of a hypothetical cohort of 12-year-old girls who were either administered the cervical cancer vaccine or who received the current standard of care from adolescence to death. For each strategy, the model incorporated probabilities of occurrence and progression of high-risk HPV, of squamous intraepithelial lesions (SIL) and of cervical cancer, as well as the probability of death, quality of life and costs associated with the corresponding health states. Every year, each person is at risk of developing high-risk HPV, SIL or cervical cancer. Over time, an infected woman's HPV infection can regress, persist or progress into SIL. High-grade SIL may possibly progress to cervical cancer. In addition to being at risk for death caused by cervical cancer, all women are still at risk for age-specific death that is unrelated to cervical cancer.

We assumed that girls with and without vaccination would receive the same standard of care that is currently being implemented, which includes routine papanicolaou (Pap) tests for compliant women every year starting from 30 years of age. At each screening event, cervical lesions are detected based on the sensitivity of the screening test [31,32]. Follow-up and/or treatment will take place depending on the type of detected lesion with a certain probability of success.

### Model parameters and base case assumptions

Transition probabilities for the hypothetical cohort from one clinical state to another over time were derived from published papers, reports or expert opinions. Whenever possible, data specific to Taiwan were used. Detailed information is provided in Table 1 which depicts the base case value, range for sensitivity analysis and data source for input parameters. The base case value represents our best estimate for each variable.

**Table 1:** Input parameters and sources*

Parameters	Base case value	Range for sensitivity analysis	Data source
**Vaccine variables**			
Vaccine efficacy, %	75	50-100	[10,12,46-48]
Vaccine coverage, %	100	30-100	Assumed
Age for starting vaccination, year	12	12-36	[49]
Immunity duration, year	lifetime	10-lifetime	[46,47]
Booster shot compliance, %	70	30-100	Assumed
**Screening variables**			
Age for starting cervical screening, year	30		[1,39]
Screening interval, year	1	1-5	[1,39]
Screening compliance, %	15-30	0-70	[1,39]
Pap test sensitivity for SIL	0.60	0.40-0.80	[31,32]
Pap test specificity for SIL	0.97	0.95-0.98	[31,32]
**Costs, US$**			
Vaccine cost (3 doses)	364	273-455	Assumed
Booster shot cost	121	91-152	Assumed
Cost of Pap test	16	12-20	[41]
Cost for a false-positive SIL	66	50-83	[41]
Cost of treatment for cervical cancer	10 000	7 500-12 500	[41]
Cost of treatment for high-grade SIL	245	183-306	[41]
**Utilities**			
Normal population	1		Assumed
Diagnosed SIL for 1-year	0.97	0.80-1	[13,40]
Cervical cancer	0.70	0.25-1	Assumed
Cervical cancer, follow-up	0.95	0.90-1	Assumed
**Transition probabilities**			
Incidence of high-risk HPV infection	0-0.09	0.5-2 × base case	[6-9]
HPV infection resolving	0.03-0.46	0.67-1.5 × base case	[33-38]
Developing low-grade SIL from high-risk HPV infection	0.065	0.05-0.08	[50-59]
Low-grade SIL regressing	0.027-0.142	0.67-1.5 × base case	[13,54-58,60]
Low-grade SIL regressing to previous HPV infection state, given regression occurs	0.10	0-0.20	[13,54]
Developing high-grade SIL from low-grade SIL	0.005-0.400	0.67-1.5 × base case	[50-58]
High-grade SIL regressing	0.037-0.058	0.67-1.5 × base case	[13,54-58,60]
High-grade SIL regressing to well state, given regression occurs	0.45	0.40-0.50	[13,54]
High-grade SIL regressing to previous HPV infection state, given regression occurs	0.05	0-0.10	[13,54]
High-grade SIL regressing to low-grade SIL, given regression occurs	0.50	0.40-0.60	[13,54]
Developing cervical cancer from high-grade SIL	0.038	0.03-0.06	[50-58]
Annual probability of developing symptoms with undiagnosed cervical cancer	0-1		[13,54,61,62]
Cervical cancer mortality	0.0024-0.3334	0.67-1.5 × base case	Estimated by the National Cancer Registry of Taiwan
**Treatment variables**			
Treatment efficacy, given high-grade SIL, %	95	90-100	[13,63,64]
HPV infection persists, given effective treatment of high-grade SIL, %	10	0-25	[13]
**Other variables**			
Discount rate, %	3	0-5	[43]
Cycle length, year	1		Assumed

*HPV denotes human papillomavirus; SIL, squamous intraepithelial lesion. All probabilities are annual unless otherwise noted.

#### HPV infection

The natural history of HPV infection is complex, and clearance or persistence of infection, together with progression to SIL, differ depending on the genotype of HPV, patient characteristics and study design. To simplify the procedure, we only classified the HPV genotypes into high- and low-risk. Our age-specific estimates for incidence, progression and regression were averages for all types of oncogenic HPV (such as HPV-16, 18, 31, 33, 35, 39, 45, 51, 52, 53, 56, 58, 59, 68 and 70) based on the population prevalence data [6-9]. In our base case analysis, the annual incidence infection began at age 15, peaked at age 20 and dropped off after age 35. Given HPV infection, regression rates were highest for women < 25 years (46%/yr) and lowest for women > 50 years (3%/yr), reflecting an observation of more persistent infections in the older age group [33-38].

#### Cervical cancer

In the model calibration process, the transition probabilities for progression from high-risk HPV infection to low-grade SIL, from low-grade to high-grade SIL and from high-grade SIL to cervical cancer varied within valid ranges derived from published papers to fit the model-predicted incidence rates of cervical cancer with data taken from the National Cancer Registry of Taiwan

The probability of diagnosing asymptomatic cervical lesions is a function of a woman's likelihood of receiving a cervical screening and on the sensitivity and specificity of this test [31,32]. The Taiwanese government launched a nationwide cervical screening program in July of 1995, in which annual Pap smear screenings were offered to women aged over 30 years. According to current reports in Taiwan, the annual compliance rate for Pap testing is approximately 30% by age 60, which proportionately declines to 15% at age 70 or older [1,39].

The National Cancer Registry of Taiwan provided us with the average survival function of invasive cervical cancer, which does not differentiate at different clinical stages. From 1990 to 2005, the registry collected a total of 39,470 cases, which were linked with our National Mortality Registry between 1990 and 2007 to determine if the patient was still alive. These follow-up data provided us with detailed survival rates up to 18 years and were used in this simulation, and if a patient survived more than 15 years, we assumed that she was in a remission state and would not die of cervical cancer.

#### Quality of life

Utilities are a measure of the quality of life rated on a scale from 0 to 1, where 0 represents death and 1 represents ideal health. Undiagnosed HPV infection and cervical lesions were considered to be asymptomatic with no decrease in utility. Diagnosed low- and high-grade SIL were assigned a lower utility (namely, 0.97) for a 1-year duration [40]. Oncogenic HPV infection can remarkably affect the quality of life for a woman with cervical cancer. For invasive cervical carcinoma, a woman's utility was assumed to reduce down to 0.70 after diagnosis to reflect the severity of her disease and its effects on her quality of life. Follow-up of cervical cancer was assigned a moderate utility (0.95) once cancer went into remission. The chosen values responded to our expert criteria.

#### Costs

Only direct medical costs are considered in this study, which include the costs associated with the health care items reimbursed by the National Health Insurance (NHI) and the out-of-pocket payments such as outpatient registration fees, some drug charges or medical equipment expenses not covered by the NHI. Pap testing costs were US$16 per test. The cost of treatment for SIL or cervical cancer was based on cost of initial colposcopy and biopsy, therapy, and subsequent follow-up. These costs were estimated by published literature of Tang et al. (2009) [41], expert opinions and official tariff lists of the NHI. The vaccination cost for three doses was assumed to be US$364, which include the cost of the HPV vaccine itself, personnel, and administration. All costs were reported in 2009 US dollars with the exchange rate of 33 New Taiwan dollars to US$1.

#### Vaccine characteristics

We initially assumed the vaccine coverage rate to be 100% in the base case situation, i.e., all women received the required three doses within 1 year. Moreover, for our base case analysis, the vaccine was assumed to confer lifetime immunity against acquiring new infections by HPV-16 and HPV-18. Because vaccine longevity is uncertain, the waning of vaccine protection over time becomes an important factor that could not be avoided. We evaluated the diverse waning scenarios that required booster shots with different compliance rates in sensitivity analysis. In the base case setting, vaccine efficacy against oncogenic types was estimated at 75%. We examined a wide range of vaccine efficacy (from 50% to 100%) to allow for further development of HPV vaccines and to deal with the possibility of lower coverage in Taiwan, where the prevalence of HPV-16 and HPV-18 infection in cervical cancer could be lower than 70% [9,42].

### Outcome measures

We expressed our results in terms of the number of cervical cancer cases prevented and deaths avoided, as well as the life-years and quality-adjusted life-years (QALY) gained over a lifetime. The incremental cost-effectiveness ratio (ICER) was calculated as the accumulated total cost difference divided by the QALY gained per woman by adding vaccination to existing screening. The economic analysis adopted a 3% annual discount rate for future costs and outcomes, which converts values that will occur in the future to their present value.

### Sensitivity analysis

Sensitivity analyses were performed to account for important model assumptions and uncertainties including the vaccine characteristics, adherence to cervical screening, costs or health utilities for various conditions, parameters related to the natural history of disease, discount rate, etc; we also examined the impact of starting vaccination at different ages on the cost-effectiveness ratio for HPV vaccine in sensitivity analysis. The ranges for costs were varied from minus 25% to plus 25% of the base case estimate. For clinical variables, our ranges for sensitivity analysis represented our judgment of the uncertainties and/or variations likely to be encountered in clinical practice, based on both the literature and the opinions of experts (Table 1).

## Results

### Model validation

**Figure 2:**
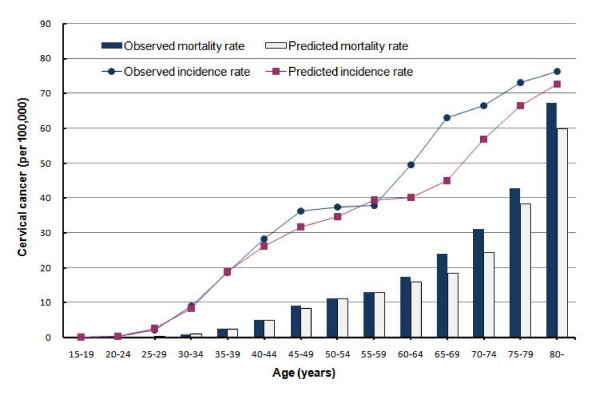
**Calibration results of age-specific incidence and mortality of cervical cancer**. The circles and bars represent the observed cancer incidence and mortality from the National Cancer Registry of Taiwan, respectively. The squares and hollow bars represent the predicted cancer incidence and mortality by the Markov model in which the current practice of cervical screening was applied from 30 years of age without vaccination.

Due to the main interests of this study and the certainty of data sources, cervical cancer incidence and mortality were chosen as primary endpoints for the model calibration process of matching the outputs in the current practice arm without vaccination of the model to observed cancer statistics. The model predicted the incidence rate for cervical cancer would be 21.1 per 100,000 females 12 or older, given the assumption that women would receive cervical screening with compliance rates of the current practice. Predicted cervical cancer incidence showed good correspondence with observed data from the National Cancer Registry of Taiwan between 2001 and 2005 that the overall incidence of cervical cancer cases was 22.7 per 100,000 females aged over 10 years (Figure 2). Moreover, the predicted HPV prevalence and cervical cancer mortality were also fit reasonably well to the observed epidemiological data, with the exception of a slightly lower mortality for ages over 65. The predicted cervical cancer mortality would be 7.2 per 100,000 females 12 or older, while the observed overall cervical cancer mortality was 7.8 per 100,000 people for women aged over 10 years.

### Base case analysis

In our base case analysis, the administration of HPV vaccine could reduce 73.3% of the total incident cervical cancer cases from 1,773 to 473 per 100,000 women and lessen 73.4% of cancer deaths from 710 to 189 per 100,000 women over the lifetime of the cohort of 12-year-old girls. On average, their life expectancy would be improved by 4.9 days or 0.024 QALY. Adding an HPV vaccine was more expensive than current practice, with an overall increase in estimated lifetime discounted cost of US$324. The incremental cost-effectiveness ratio based on this model was US$13,674 per QALY gained (Table 2).

**Table 2:** Health and economic outcomes of HPV vaccination, discounted

Outcome	No vaccination	HPV vaccination
Cost, US$	129	453
Incremental cost, US$		324
Life expectancy, years	28.830	28.844
Incremental life expectancy, days		4.9
Quality-adjusted life expectancy, years	28.816	28.840
Incremental quality-adjusted life expectancy, days		8.7
Incremental cost-effectiveness ratio		
US$/life year		23 939
US$/quality-adjusted life year		13 674

HPV denotes human papillomavirus

### Sensitivity analysis

**Figure 3:**
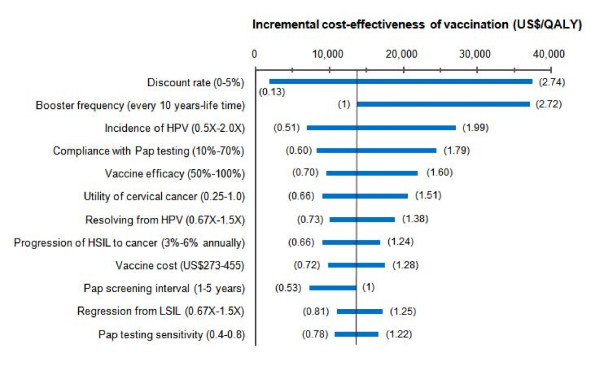
**One-way sensitivity analyses on the incremental cost-effectiveness ratio (ICER)**. The range of input parameter for the sensitivity analysis is indicated in the parentheses on the left of the vertical axis. The vertical line represents the ICER under base case assumptions. The numbers in brackets alongside the bar represent the ratio between the maximum value (right) and the minimum one (left) in sensitivity analysis respectively over the base case ICER.

On the basis of our sensitivity analyses of various parameters, the model suggested that the ICER of adding vaccination strategy, as compared to the current practice, is most sensitive to variations in discount rate, vaccine immunity longevity or booster frequency, incidence of high-risk HPV infection, compliance with Pap testing, vaccine efficacy and in quality of life for cervical cancer. Changes in these parameters could result in wider variations of the ICER, as depicted in Figure 3.

**Figure 4:**
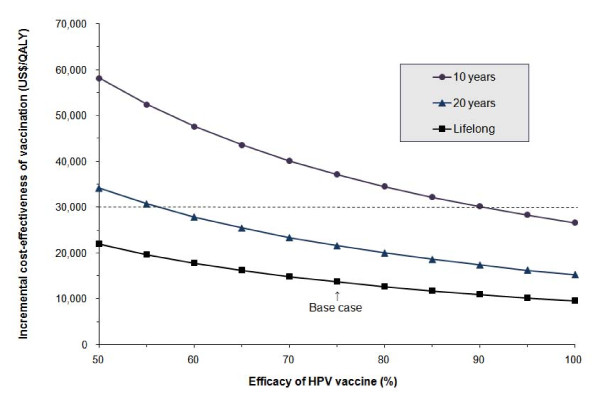
**Sensitivity analysis of the incremental cost-effectiveness of vaccination compared to the current practice, with respect to the change in human papillomavirus (HPV) vaccine efficacy and immunity longevity**. The squares represent a vaccine providing lifetime immunity to HPV-16 and HPV-18 (base case assumption). The triangles represent a vaccine that requires booster shots every 20 years to remain effective. The circles represent a vaccine that needs booster shots every 10 years to maintain effectiveness.

The incremental cost of vaccination would be usually less than US$30,000/QALY relative to the current practice when the efficacy was greater than 37%. If vaccination required a one-shot booster every 10 years, then the ICER would increase to US$37,150/QALY which multiplied by 2.7 the base case outcome under lifelong immunization (Figure 4). The ICER would remain below US$30,000/QALY if the interval of booster shots needed to maintain the immunity was over 13 years.

Vaccination cost-effectiveness, however, would be US$37,480/QALY at a discounted rate of 5% since higher discount rates augment the relative weight of the initial vaccination costs.

If every woman in Taiwan obtained a Pap test every 3 years from the age of 30, the ICER of vaccination would slightly increase to US$17,199/QALY.

## Discussion

Our analysis demonstrated that under most assumptions, the prophylactic vaccination against HPV-16 and HPV-18 had an ICER between US$7,000 and US$27,000 per QALY gained in the vaccinated adolescent girls in Taiwan. The ICER would remain below US$30,000 per QALY unless the vaccine efficacy declined to less than 38% or if the immunity waned and required booster shots every 10 years (Figure 4). If the vaccination cost could be reduced to below US$277, then the HPV vaccination would cost less than US$10,000 per QALY gained, indicating a potential for further enhancement of cost-effectiveness. Although there has been no domestic consensus on the threshold of the cost-effectiveness ratio for the National Health Insurance system to decide whether to reimburse a new medical intervention, the results of our analysis suggest that prophylactic vaccination against oncogenic HPV administered in preadolescent girls in Taiwan would be usually cost-effective based on the World Health Organization proposed criteria of 1-3 times the gross domestic product (GDP) per capita being cost-effective or less than GDP per capita being very cost-effective [43] since the GDP per capita of Taiwan was approximately US$17,082 in 2008.

In addition to the discount rate, the duration of the vaccine immunity accounted for the most influential source of variations in the ICER of incorporating HPV vaccination within our investigation (Figure 3), which is consistent with other studies [27]. Compared to the previous studies mostly performed in Western countries [16,22-26], the HPV vaccination strategy in our study appeared to be attractive in terms of a lower ICER. However, this figure of cost-effectiveness in Taiwan could be largely owing to the high prevalence of HPV infection [6-9] and lower compliance rate with cervical cytological screening [1] that resulted in higher background incidence of cervical cancer, since we employed similar assumptions of time horizon, discount rate, vaccine efficacy and lifelong vaccine protection as most of those studies. For example, as the projected incidence of cervical cancer under the current screening practice in a study from the Netherlands [26] was lower than that in our study by 3.5 times and the vaccination costs were 1.5 times more expensive, the ICER reported by them was much higher than the figure in our study (approximately 5.8 times).

Methodological differences may also account for variations in the results of different cost-effectiveness evaluations [27,44]. Although dynamic transmission model has been developed and applied [17,18,22,23,25,28], it generally requires investigators to make more assumptions on putting into parameter values related to viral transmission. As the sexual behavior in adolescents and young people in Taiwan may be different from that in western countries and the relevant data were insufficient, we took an alternative approach to adapt a simpler Markov model as previous studies [13,14,24], but more delicately adjusted the model with existing clinical and epidemiological data of cervical cancer in Taiwan. Our approach did not consider the herd immunity and the protection by HPV vaccination for genital warts or other HPV related cancers. Thus, we would underestimate the overall effectiveness of the vaccination program, which would generally make the cost-effectiveness of the HPV vaccine even more favorable if herd immunity or protection for other diseases existed [21,45].

The relatively high risk level of invasive cervical cancer in Taiwan implies the urgency to improve the compliance rate of cervical screening to the early detection of SIL and cervical cancer, even though the ICER of prophylactic vaccination would rise accordingly because the marginal effectiveness of vaccination would be diminished as improvement in cytological screening would decrease the baseline incidence of invasive cervical cancer without adding HPV vaccination. Moreover, during the model calibration process, we discovered an upward trend of cervical cancer incidence by age that reflected inadequate compliance with cervical screening among older women, particularly those older than 60 years (Figure 2). Had the cervical screening compliance for older women improved to be comparable with those of younger women, the cumulative incident cases with cervical cancer would have decreased in both cohorts with or without vaccination, while the ICER of HPV vaccine would go up slightly to US$14,120 per QALY gained.

The impact of discounting is very complex in the context of HPV vaccination. As in any economic assessment of a preventive measure with later-onset effects, the initial intervention costs and the choice of discount rate have a significant influence on the cost-effectiveness results. In general, higher discount rates would make the prophylactic vaccination strategy seem less attractive, given that the costs of the intervention are paid immediately while the benefits come back many years later. Indeed, we found the undiscounted ICER of vaccination on 12-year-old girls was US$1,820 per QALY gained, whereas the ICER significantly increased to US$37,480 per discounted QALY gained at a discounted rate of 5%.

There are limitations in this study. First, herd immunity effects were not taken into account in our model as discussed above. Second, women adherent to previous cervical screening tests tended to have better compliance with subsequent tests [1]. The preventive effects of screening could therefore be overestimated particularly for those at older ages, which in turn would underestimate the effectiveness of vaccination. Thus, the current ICER of HPV vaccine would be a conservative estimation, as the ICER should further decline if the actual compliance rates of cervical screening were adjusted with a lower coverage. Nonetheless, the conservative assessment for the ICER of HPV vaccine in our study, together with the results of other relevant research [28,29], would increase the credibility of the cost-effectiveness for a prophylactic HPV vaccination program in Taiwan.

## Conclusions

Our analysis suggested that vaccination of adolescent girls with an HPV vaccine seems to be cost-effective in Taiwan where the HPV infection rate and the incidence as well as the mortality of cervical cancer are relatively higher than those in other developed countries. Although there are still some uncertainties regarding the HPV vaccine and cervical cytological screening, our estimation of the cost-effectiveness for a prophylactic vaccine against high-risk HPV, however, appears to be robust. We have demonstrated that the ICER would usually fall below US$30,000 per QALY gained under most assumptions, which also covers a wide range of vaccination strategies and vaccine characteristics. Even in the case of favorable cost-effectiveness ratio of prophylactic vaccination against oncogenic HPV, there is still room for improvement of the compliance with Pap screening tests in Taiwan, especially for older women, because vaccination should not yet be regarded as the substitute for cytological screening. It calls attention to the importance of continuing research that investigates primary and secondary preventive measures against cervical cancer.

## Competing interests

S-NC has served on advisory boards for GlaxoSmithKline, and has been the Principal Investigator at National Taiwan University Hospital for GlaxoSmithKline clinical trial (HPV-008 Study) since 2004 up to now. All the other authors declare that they have no interests which might be perceived as giving rise to any form of bias or conflict of interest.

## Authors' contributions

All of the authors formulated the research question and design of the study. P-HL extracted the data and carried out the analyses. F-CH, P-IL, S-NC and J-DW provided intellectual input into the analyses and/or interpretation of data. C-WH participated in the analyses. P-HL and J-DW prepared the first draft of the manuscript. F-CH, P-IL, and S-NC provided content expertise and contributed to the final version of the manuscript. All authors have read and approved the submission of the manuscript to BMC Health Services Research in its present form.

## Pre-publication history

The pre-publication history for this paper can be accessed here:

http://www.biomedcentral.com/1472-6963/10/11/prepub
